# The microbiota-gut-brain axis and three common neurological disorders: a mini-review

**DOI:** 10.1097/MS9.0000000000000552

**Published:** 2023-04-01

**Authors:** Amjad Mhanna, Zuheir Alshehabi

**Affiliations:** a Faculty of Medicine, Tishreen University; b Department of Pathology, Tishreen University Hospital, Latakia, Syrian Arab Republic

**Keywords:** epilepsy, gut-brain-axis, microbiota, migraine, parkinson’s disease

## Abstract

Neurological disorders are an important cause of disability and death globally. Recently, a large body of research shows that the gut microbiome affects the brain and its conditions, through the gut-brain axis. The purpose of this mini-review is to provide a brief overview of the relationship between the microbiota-gut-brain axis in three neurological disorders: epilepsy, Parkinson’s disease, and migraine. The authors chose these three disorders because of their burdensome and great effect on health care. We live on a microbial planet. Before humans, microorganisms existed for a hundred million years. Today, there are trillions of these microbes living in our bodies, it is called human microbiota. These organisms have a crucial role in our homeostasis and survival. Most of the human microbiota live in the gut. The number of gut microbiota is much more than the number of body cells. Gut microbiota has been regarded as a crucial regulator of the gut-brain axis. The discovery of the microbiota-gut-brain axis is described as a major advancement in neuroscience because it influences the pathophysiology of several neurological and psychiatric disorders. From this, more studies of the microbiota-gut-brain axis are needed in the future, to provide a better understanding of brain disorders and so that better treatment and prognosis.

## Introduction

Highlights

- The microbiota-gut-brain axis is related to the pathophysiology of several neurological and psychiatric disorders.
- The usual therapy for refractory epilepsy may change in the future to involve microbiome-based therapeutic tools.
- The microbiota affects the etiology of Parkinson’s disease with the help of the microbiota-gut-brain axis.
- The gut microbiota and migraines are related, and through this relationship, several lines of treatment could be used.

The Earth is a microbial planet; microbes have been existing for a hundred million years before humans. The term ‘human microbiota’ refers to the trillions of microbes that live in our bodies. There is no time throughout our evolution that we lived without these tiny organisms. The microbiota is a crucial factor for our survival and its existence is essential for human health^1^. Many agents define the microbiome content diversity between individuals, such as age, genes, environment, nutrition, infections, and antibiotic usage^2^. Most of the human microbiota live in the gut^1^. The number of gut microorganisms is much more than the number of body cells. We have nearly 10^13^–10^14^ microbes in our bowels^3^. These microbes affect many systems and organs in the human body. One of these organs is the brain. Recently, the gut microbiota has been considered an important regulator of the gut-brain axis, the term that refers to a bidirectional link between the intestine and brain^4^. This connection consists of many pathways, including the immune system, peripheral nerves, and the hypothalamus-pituitary-adrenal axis^5^. The discovery of the microbiota-gut-brain axis is described as a great achievement in neuroscience because it is related to the pathophysiology of several neurological and psychiatric disorders like epilepsy, migraine, Parkinson’s disease (PD), depression, anxiety, autism spectrum, and schizophrenia^4^. In addition to noticing that there is a difference between gut microbes species in patients with neurodegenerative and neuropsychiatric diseases compared with healthy humans^6^. Herein, we choose to highlight in brief the link between the microbiota-gut-brain axis and three common neurological disorders.

## The microbiota-gut-brain axis

The bidirectional link between the gut and brain includes several pathways, such as the enteric and autonomic nerves, the hypothalamic-pituitary-adrenal (HPA) axis, the immune system, and the chemical substances^7^. The brain and the intestines are connected directly through neural connections. The most well-known one is the vagus nerve, which is extending from the brainstem to all visceral organs^8^. Another pathway is the HPA axis, which is regarded as the most significant component in the neuroendocrine system. The HPA axis regulates stress responses and is considered an essential pathway in the microbiota-gut-brain axis. According to animal studies, the HPA axis could alter the microbiome composition and vice versa^1^. The immune system impacts both the gut and the brain, and it is also impacted by them. The gut microbiota has a critical role in immune system physiology. For example, the healthy function of microglial cells in the brain is affected by the gut microbiome^8^. In addition to recent pathways, many chemical substances contribute to the gut-brain axis. Short-chain fatty acids (SCFA) are an example of these substances^4^. SCFA produces by gut microbiota and directly affects the brain, by influencing neural plasticity, epigenetics, and gene expression^8^. Neurotransmitters are another example. The intestinal microorganisms could modulate the levels of serotonin, noradrenaline, dopamine, glutamate, and gamma amino butyric acid (GABA). Thus, the gut microbiota impacts brain function^4^.

**Figure 1:**
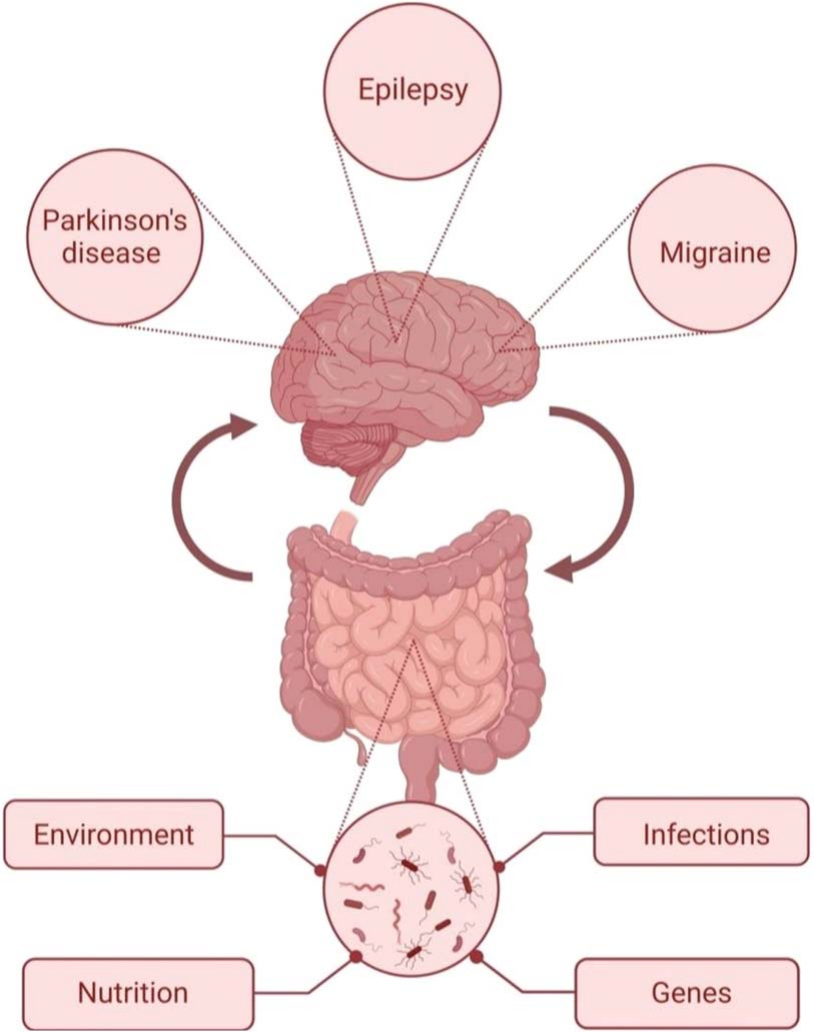
The microbiota-gut-brain axis and three common neurological disorders: epilepsy, Parkinson’s disease, and migraine. In addition to many factors related to gut microbiome diversity, such as genes, environment, nutrition, and infections. *Source*: This figure has been created with BioRender.com.

Several factors affect the gut microbiota composition, such as age, genes, diet, environment, infections, and antibiotics usage (Fig. 1).

The age of the host has a clear impact on the microbiota composition^9^. The microbiota composition varies between newborns and adults, it is also continually changing with age^9^,^10^. Additionally, the gut microbiota is strongly influenced by genes. For instance, the microbiome is related to many genes contributing to the innate immune system of the host. Nutrition also influences microbiota’s diversity, it could increase or decrease particular species.

Furthermore, antibiotics usage could negatively alter the gut microbiome, according to the type of antibiotics and the duration of the treatment^10^.

Many studies showed that early childhood antibiotic usage may be linked to the etiology of several neurocognitive diseases^11^. Host infections could also affect the composition of the microbiota. For example, during the coronavirus disease 2019 pandemic, researchers noticed that there is an alteration in fecal microbiota diversity. Hence, altering the gut flora has shown a considerable improvement in the symptoms^9^.

## The microbiota-gut-brain axis and epilepsy

Over 70 million individuals worldwide suffer from epilepsy, which is one of the most prevalent brain disorders^12^. The pathophysiology of epilepsy is complex and the percentage of idiopathic cases is 60%^6^. We know that there is a loss of balance between the excitation and inhibition of neurons, which leads to the appearance of spontaneous seizures^4^.

However, pharmaceuticals are the most widely used anti-epileptic therapies, but seizures cannot be managed by medication therapy in more than 30% of epileptic patients. This phenomenon is called refractory epilepsy^6^. There is a type of diet that may be useful for those patients, it is called the ketogenic diet. It is a diet that contains a high level of fat and a low level of carbohydrates^4^,^13^. This diet is a common therapy recommended to epileptic patients and widely used as a treatment for epilepsy in a lot of countries across the world^1^,^13^. This clinical observation provides evidence that the gut and epilepsy are related^6^. The exact mechanism by which the ketogenic diet affects epilepsy is still unclear^14^. It seems that this diet has an important influence on the composition of microbiota^1^. According to several recent studies, the composition of the fecal microbiota differs statistically between healthy individuals and epileptic patients and also differs between patients with epilepsy before and after ketogenic diet therapy.

Small sample size studies showed differences in gut microbiota between epilepsy patients and healthy controls. Another study showed more diversity of gut microbiota in healthy people compared to refractory epilepsy patients.

Many researchers found that there is dysbiosis in the gut microbiome in epileptic patients. Therefore, the diagnosis and treatment of epilepsy may be significantly impacted by the gut microbiota.

The pathophysiology of epilepsy may include the immunological and inflammatory pathways in the gut-brain axis.

The gut microbiota modulates the immune system and inflammatory processes, which in turn controls the development of epileptic seizures.

Additionally, it is well-known that neurotransmitters have a great role in epilepsy pathogenesis. They are also strongly impacted by the gut microbiota. Many neurotransmitters might be produced by these microorganisms. The gut microbiota has an impact on the glutamine-glutamate-GABA cycle and controls the expression of GABA receptors in certain parts of the brain^6^.

The microbiota-gut-brain axis may be a promising goal to expand our understanding of the mechanism by which the ketogenic diet controls epilepsy^13^. And the usual therapy for refractory epilepsy may change in the future to involve gut microbiota remodeling^6^.

## The microbiota-gut-brain axis and Parkinson’s disease

PD is a common progressive neurodegenerative disease with no fully understood etiology^15^, and no healing treatment until today^16^. It affects 7 to 10 million individuals in the world^15^ and has shown a sharp increase in incidence and prevalence in the last few years^17^. The symptoms of PD are divided into motor and nonmotor symptoms^4^. The motor features include tremors, rigidity, and bradykinesia. The nonmotor features involve a lot of symptoms, for example, depression, sleep problems, and gastrointestinal symptoms^15^,^18^. It is important to notice that over 80% of PD patients suffer from digestive dysfunction^15^,^19^.

The pathophysiology of the disease in brief is the following: there is a progressive loss of dopaminergic neurons in the substantia nigra pars compacta^4^, there are intracellular inclusions called Lewy bodies, and these bodies contain a protein called alpha-synuclein^15^.

Many studies demonstrate that the gut-brain axis is concerning for alpha-synuclein pathology^4^. Alpha-synuclein protein is found in both the central nervous system and the enteric nervous system^4^,^20^ and is related to digestive symptoms of PD^20^, which often start before the appearance of typical motor symptoms^19^. These observations suggest that the pathophysiology of PD may initiate in the intestine^1^,^20^. The microbiota may have a role in the impact of alpha-synuclein through progressing the disease^1^. The microbiota affects the etiology of PD with the help of the microbiota-gut-brain axis^19^. Various clinical research has recently provided evidence that the composition of the gut microbiota is different in people with PD^15^. The metabolism of mitochondria may be impacted by the gut microbiome, and mitochondrial dysfunction has a role in PD neurodegeneration^21^.

Moreover, protection against neuronal loss has been noticed in germ-free animals^17^.

However, monitoring the change in the bowel microbiota and its metabolites may be helpful in the diagnosis of PD earlier^22^. Additionally, microbial treatments like probiotics and fecal microbiota transplantation can help with both motor and nonmotor symptoms of disease^19^,^22^.

## The microbiota-gut-brain axis and migraine

Migraine is a frequent problem in neurology^23^. It is considered the most common neurological condition seen in primary care^24^, and the second most common reason for disability in the world^24^,^25^.

Migraine is a very burdensome disease experienced by about 18% of females, 6% of males and 2% of people worldwide suffer from chronic migraine^24^. The headache in a migraine attack is usually unilateral, pulsing, exacerbated by physical activity, and commonly accompanied by the following symptoms: vomiting, nausea, phonophobia, and photophobia^25^.

In addition to the above, there is a relationship between migraine and many gastrointestinal disorders^26^, like celiac disease, irritable bowel syndrome, and helicobacter pylori infection^27^,^28^.

However, it is still unclear what causes migraines exactly^28^. Various evidence suggests that the microbiota-gut-brain axis has an important role in the pathophysiology of disease^28^,^29^. Compared to healthy people, the gut microbiota of patients with migraine is different^30^. The microbial alteration in the gut may cause many changes. For example, increasing the release of calcitonin gene-related peptide, and modulating the signals of tumor necrosis factor alpha in the trigeminal nerve system. These changes have a great impact on the etiology of migraine^26^,^29^,^30^.

In both human and animal research, there is a clear relation between the vagus nerve and migraine pathophysiology. The stimulation of this nerve could reduce migraine pain. Additionally, SCFA, which is produced by gut microbiota might be useful in treating migraine patients^30^.

It is well-established that gut microbiota and migraines are related^26^. And through this relationship, several lines of treatment could be used. For instance, patients on a ketogenic diet report less frequent and more manageable migraine episodes^31^. Moreover, probiotics may be another useful option in controlling migraine^26^.

## Conclusion

It is clear today that neurological disorders are strongly related to the gut and its microbiome. Epilepsy, PD, and migraine are three among the numerous examples of this relationship. Both microbiome-based diagnostic tools and microbiome-based therapeutic tools could be useful. This paper provides a brief overview of many points: the pathways between the gut and brain, the role of the gut microbiome in three common brain disorders (epilepsy, migraine, and PD), and the possibility of treating these disorders according to recent findings.

In the future, more research is needed to detect the exact cause of these disorders and through studying the microbiota-gut-brain axis, better treatments might be discovered.

## Ethical approval

Not applicable, because this article does not contain any studies with human or animal subjects.

## Consent

This article is a narrative review.

## Sources of funding

This research received no specific grant from any funding agency in the public, commercial, or not-for-profit sectors.

## Author contribution

All authors contributed in all the phases of preparing the paper.

## Conflicts of interest disclosure

The authors declare that there is no conflict of interest.

## Research registration unique identifying number (UIN)

It is a literature review; not a clinical trial.

## Guarantor

Prof. Dr Zuheir Alshehabi.

## Provenance and peer review

Not commissioned, externally peer-reviewed.

## Authorship

All authors attest that they meet the current ICMJE criteria for authorship.
